# Case report: Desmoplastic ameloblastoma

**DOI:** 10.4103/0971-3026.35819

**Published:** 2008-02

**Authors:** Rajul Rastogi, Harsh Jain

**Affiliations:** Yash Diagnostic Center, Yash Hospital and Research Center, Moradabad, UP - 244 001, India; 1 Dept. of Oral and Maxillofacial Surgery, Kothiwal Dental College and Research Center, Moradabad, UP - 244 001, India

Ameloblastomas are tumors arising from the odontogenic epithelium.[1] Despite their locally destructive nature, they are considered benign.[2] They are the commonest neoplasms affecting the jaws.[3] The most common types of ameloblastoma are the follicular and plexiform varieties, followed by the acanthomatous and granular cell types. Uncommon variants include desmoplastic, basal cell, clear cell, keratoameloblastoma, and papilliferous keratoameloblastoma. When the desmoplastic type coexists with other types, it is called a ‘hybrid’ ameloblastoma.[4]

There are significant anatomic, histopathologic, and radiologic differences between desmoplastic ameloblastomas and the classic type.

## Case Report

A 45-year-old lady presented with a painless hard swelling in the anterior part of the lower jaw of 6 months’ duration. Clinical examination revealed a bony hard swelling arising from the lower jaw, with the intraoral examination showing a large, hard, nontender mass on the anterior mandible, covered by red, intact, and mobile mucosa. The lesion extended from the right premolar region to the left canine region. No lymphadenopathy or fistulae were present. The involved teeth were vital and slightly displaced lingually. The past medical history was unremarkable.

**Figure 1:**
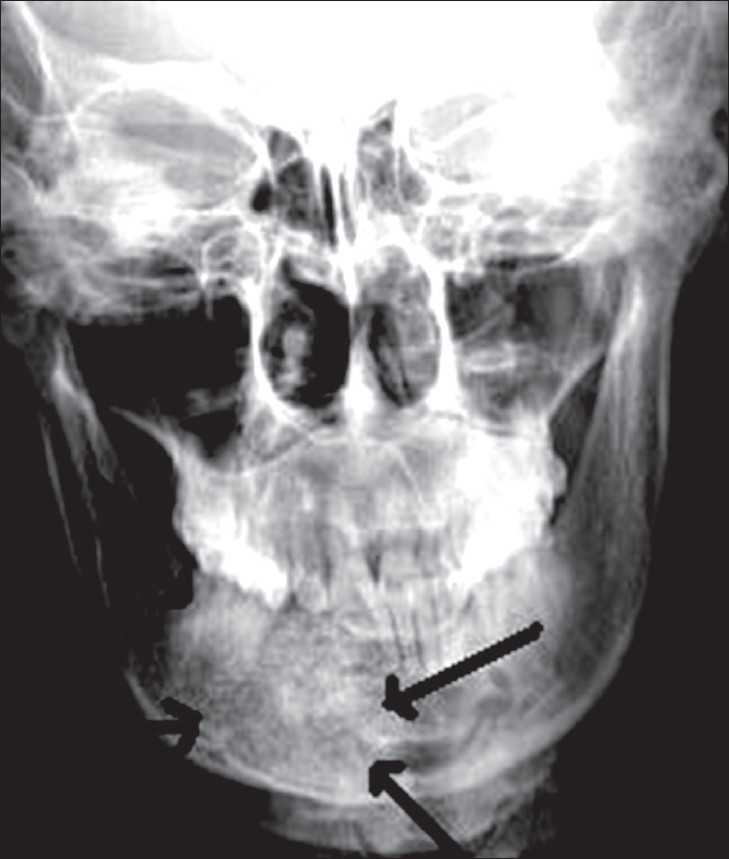
Radiograph of the mandible (PA view) shows a diffuse, radiodense lesion with a honeycomb appearance in the anterior mandible on the right, with lingual displacement of the adjacent teeth and possible extension across the symphysis

A frontal radiograph of the mandible revealed a diffuse, ill-defined, predominantly radioopaque lesion interspersed with fine radiolucent areas, producing a honeycomb appearance; this arose from the body of the mandible in the region of the right incisors, canine, and premolars [Figure 1].

**Figure 2:**
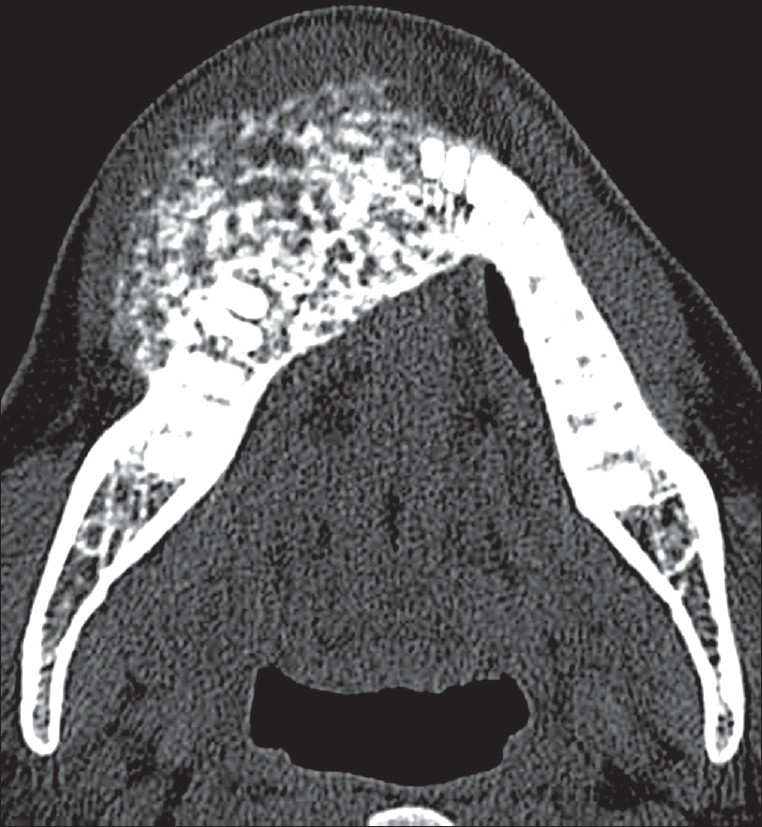
Axial CT of the mandible with bone window settings, shows an expansile, solid, mixed radiodense-radioopaque lesion with poorly defined margins on the right anteriorly, with destruction of the cortices and extension into the soft tissues and across the symphysis as well

**Figure 3:**
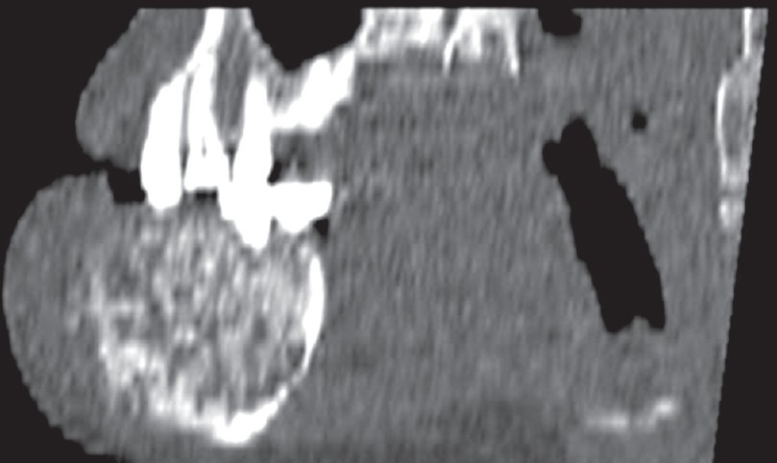
Sagittal MPR CT image of the mandible with bone window settings shows an expansile, solid, mixed radiodense-radioopaque lesion with poorly defined margins in the anterior mandible, with destruction of the cortices and extension into the soft tissues

A plain CT scan clearly showed the internal characteristics and extent of the lesion. It revealed a solid, expansile, ill-defined, radiodense mass intermixed with radiolucent areas, arising from the anterior part of the body of the mandible and extending from the right incisor to the right premolar region, as well as across the symphysis. The lamina dura of the affected teeth and the buccal and lingual cortices were destroyed [Figures 2 and 3].

Based on the radiological appearances, the diagnostic possibility of either a fibro-osseous mass or an ameloblastoma (desmoplastic variant) was suggested. The patient underwent an incisional biopsy, which showed a desmoplastic ameloblastoma. A right hemimandibulectomy across the midline was then performed with a transitional prosthesis.

## Discussion

The desmoplastic ameloblastoma is a rare and infrequent tumor, characterized histologically by marked stromal desmoplasia.[5] The common age of presentation is from the third to the fifth decades. Among patients, men outnumber women.[6] Demographic studies have revealed that the highest incidence of this tumor is in patients of the Japanese race.[6]

The majority of desmoplastic ameloblastomas occur in the mandible, commonly in the anterior part.[6] The classic type, in contrast, is commoner in the posterior mandible. Clinically, maxillary lesions are more dangerous than mandibular ones as they can invade the adjacent sinus and orbit and involve vital structures. Besides, the thin maxillary bone is a weak natural barrier for tumors as compared to the thicker mandible.[7]

Radiographically, a desmoplastic ameloblastoma is seen either as an ill-defined mass containing osteolytic and sclerotic areas or as multifocal radiodense flecks within a radiolucent background, resembling a honeycomb. This is because of the infiltration of the tumor cells into the adjacent marrow spaces, with simultaneous vigorous osteoblastic activity.[8] The tumor may be unilocular or multilocular.[6] CT scan can delineate the internal structure of the lesion more accurately and is particularly helpful in determining its margins and extension into adjacent structures.[4]

MRI shows heterogeneous low to intermediate signal intensity on T1W images, heterogeneous high signal intensity on T2W images, and strong enhancement on post-gadolinium T1W images.[9] MRI can clearly differentiate between solid and cystic components.[10]

On histopathology, desmoplastic ameloblastoma reveals small areas and thin cords of odontogenic epithelium distributed between dense, fibrous connective tissue.[5] Regions of mature lamellar bone may be seen and invasion may be demonstrated.[5] This histological finding may indicate the potential for local invasion and accounts for the diffuse appearance on radiographs. Desmoplastic ameloblastoma is therefore considered more aggressive than other common variants of ameloblastoma.[5]

As it is almost impossible to find the exact interface between the lesion and normal bone, it is difficult to cure these tumors surgically.[11] Since desmoplastic ameloblastomas tend to infiltrate between bone trabeculae, curettage often leaves islands of tumor within the bone, which eventually leads to recurrences. Therefore, block excision is the most widely accepted form of treatment.[7]

Thus, desmoplastic ameloblastoma should always be considered in the differential diagnosis of a mixed radiodense-radiolucent lesion with diffuse borders in the anterior premolar region of the jaws. Other possibilities include fibro-osseous lesions like cemento-ossifying fibroma, cementoma (intermediate stage), osteitis, cementoblastoma, fibrous dysplasia, calcifying odontogenic cyst, etc. A definitive diagnosis prior to surgery requires histopathology, to aid proper surgical management.
